# LncRNA PROX1-AS1 promotes proliferation, invasion, and migration in papillary thyroid carcinoma

**DOI:** 10.1042/BSR20180862

**Published:** 2018-09-12

**Authors:** Yanyan Shen, Erjie Xia, Adheesh Bhandari, Xiaohui Wang, Guilong Guo

**Affiliations:** Department of Thyroid and Breast Surgery, The First Affiliated Hospital of Wenzhou Medical University, Wenzhou, Zhejiang, PR China

**Keywords:** invasion, LncRNA, migration, Prox1-as1, PTC, proliferation

## Abstract

Evidence has been provided that long noncoding RNAs (LncRNAs) play major roles in affecting essential physiological processes, and many of which seem to have functional roles in tumorigenesis and progression. However, the intrinsic molecular mechanism of LncRNAs acting on papillary thyroid carcinoma is not well understood. In the present study, we found that PROX1-AS1 levels were obviously increased in thyroid cancer cells compared with the normal thyroid epithelial cells. Knockdown of PROX1-AS1 gene expression by siRNA could inhibit cell proliferation. Subsequently, we also observed that silencing PROX1-AS1 might inhibit invasion and migration of thyroid cancer cell lines via modulating the expression of epithelial–mesenchymal transition related proteins. In conclusion, our study indicated that LncRNA PROX1-AS1 could promote papillary thyroid carcinoma development and might serve as a potential targeting marker for papillary thyroid carcinoma.

## Introduction

Papillary thyroid carcinoma (PTC) is the most common type of the thyroid cancer, around taking up 60–70 percent of all thyroid malignancy, and in the fourth and fifth decades of a women life, it may often happen [1]. Even though its incidence currently is higher than before, it still has a great prognosis, with a 10-year survival rate above 90 percent [1,2]. In spite of high incidence and low mortality rate, further researches should be launched to find out the underlying mechanisms of PTC, so as to search more novel potential targets for PTC treatments.

It is estimated that more than 70% human genome is transcribed into RNA; however, less than 2% of the human genome encodes protein [3,4]. Long noncoding RNAs (LncRNAs) are a series of non-protein-coding transcripts owing to shortage of open reading frames, and they are longer than 200 bp that are encoded by the mammalian genome [5]. Lots of studies have suggested that LncRNAs may function in diverse vital physiological and pathological processes [6,7] and play crucial roles in cancer development and metastasis because of its regulation. For example, H19 is one of the first reported long noncoding RNAs. It is up-regulated in the breast, respiratory and brain tumors, and plays a part in trans to suppress the levels of a number of imprinted genes [8,9]. Lu et al. [10] discovered that P21-associated ncRNA DNA damage-activated (PANDAR) was related to invasion, lymph node metastasis in colorectal cancer, which would result in poor overall survival.

With the development of gene sequencing technology, more and more longer noncoding RNAs have been discovered. In order to find some new biomarkers in thyroid cancer, we carried out the whole genome of 19 pairs of thyroid cancer and adjacent non-cancerous tissues in our unpublished study. In the result, we found that PROX1 antisense RNA 1 (PROX1-AS1) is extremely up-regulated in thyroid cancer compared with adjacent non-cancerous tissues. Thus, we chose PROX1-AS1 for the further cell experiment. PROX1-AS1 is transcribed from the antisense strand of PROX1 and located in human chromosome 1q322.3, a transcript of 3399 bp. Previous studies have found that PROX1-AS1 variants could increase the risk of osteonecrosis in children under age 10 treated for acute lymphoblastic leukemia, which possibly acted by modifying lipid trafficking within the compartment of the bone marrow or by increasing plasma lipids [11,12]. However, the effect of PROX1-AS1 in PTC is unclear. The purpose of our research was to identify the role of PROX1-AS1 related to the development and metastasis of PTC. Moreover, the molecular mechanism by which PROX1-AS1 regulated in PTC cells was studied.

## Materials and methods

### Cell lines culture

Human thyroid carcinoma cell lines (KTC1, TPC1, and BCPAP) were provided by Prof. Mingzhao Xing of the Johns Hopkins University School of Medicine, Baltimore, MA, U.S.A. Our cells were cultured in RPMI1640 medium (Invitrogen, Carlsbad, CA, U.S.A.) supplemented with 10% fetal bovine serum (FBS; Invitrogen) in a humidified 37°C incubator (Thermo, Waltham, MA, U.S.A.) with 5% CO_2_.

### Cell transfection

The BCPAP and TPC1 cells were transfected with siRNA (Shanghai, China) and Lipofectamine RNAiMAX transfection reagent (Invitrogen). All cell lines were plated in six-well plates before transfection. PROX1-AS1 were silenced with 10 μl of (TPC1) or 5 μl of (BCPAP) siRNA and 4 μl of RNAiMAX for 48 h. The siRNA sequences used in our research are: PROX1-AS1 siRNAs target the following sequences: PROX1-AS1 siRNA-1, Forward 5′-GCAGCAGAUUUACGGCAAATT-3′ and Reverse 5′-UUUGCCGUAAAUCUGCUGCTT-3′; PROX1-AS1 siRNA-2, Forward 5′-GCCUGGAUAUGUUGUAGUATT-3′ and Reverse 5′-UACUACAACAUAUCCAGGCTT-3′.

### RNA extraction and qRT-PCR

All cells RNA was extracted and purified using TRIzol reagent (Invitrogen) and then reverse transcribed by Thermo Scientific RT reagent Kit (Thermo Scientific) according to the manufacturer’s protocol. qRT-PCR was performed using Thunderbird SYBR qPCR Mix (Toyobo, Osaka, Japan) in the Applied Biosystems 7300 Real-Time PCR System (Applied Biosystems, Foster City, CA). The LncRNA transcript levels were measured using the comparative cycle threshold (*C*_T_) (2^−ΔΔ*C*^_T_) method and normalized with the GAPDH levels. The primer sequences were listed followed: PROX1-AS1 Forward: 5′-CTAGTTAGCAGGGGCAGCAC-3′ and Reverse: 5′-AACAGAGAGGCGTGGAAGAA-3′. GADPH Forward: 5′-GTCTCCTCTGACTTCAACAGCG-3′ and Reverse: 5′-ACCACCCTGTTGCTGTAGCCAA-3′.

### Protein extraction and Western blot analysis

The cells were collected and lysed in lysis buffer (Beyotime, Shanghai, China). And then aliquots of the lysate were separated by 10−15% SDS-polyacrylamide gel electrophoresis (SDS-PAGE) and transferred to PVDF membranes (Millipore, Billerica, MA). Blocking PVDF membranes with 5% nonfat milk and incubating the membranes overnight at 4°C with primary antibodies to target protein. Then washed with TBST three times and incubated with the anti-rabbit IgG or anti-mouse IgG (Abcam, Cambridge, MA) for 1 h at room temperature. The bands were detected by the method of scan the absorbance after chemiluminescence. Primary antibodies were as follows: N-cadherin, Vimentin, E-cadherin (Abcam, U.S.A.) and human GAPDH (Sigma, U.S.A.).

### Colony forming assay

TPC1 and BCPAP cell lines transfected with si-NC or si-RNA were placed in six-well plates and maintained in 10% FBS for 7–10 days. Then fixed with 4% paraformaldehyde and stained with 0.4% Crystal Violet solution, finally photographed.

### CCK-8 proliferation assay

Cell Counting Kit-8 assay (CCK-8, Beyotime) was used to detect the effect of PROX1-AS1 on PTC cell growth. Cells were harvested 24 h after infection by siRNA, then the transfected cells were seeded on 96-well plates. Ten microliters of the CCK-8 solution was added to each well and incubated at 37°C for another 3 h. The results were quantitated using a test wavelength of 450 nm.

### Cell invasion and migration assays

According to the protocol, 24 h after transfection, trypsin was used to digest cells and then collected TPC1 and BCPAP cells with medium supplemented with 10% FBS. Transfected cells or control cells were cultured into the upper Transwell chamber for invasion or migration assay. The cells were allowed to migrate for 24 h toward the lower chamber that contained 600 µl of medium supplemented with 20% FBS. After incubation, the membranes were fixed in 4% paraformaldehyde and stained with 0.4% Crystal Violet solution for 20 min. TPC1 and BCPAP cells invasion or migration ability were assessed by the number of cells that had invaded or migrated through the membranes. Five random fields of view were chosen, and meanwhile, the images were captured under microscope magnification (×20). Experiments were carried out independently in triplicate.

### Statistical analysis

Using SPSS 23.0 software (SPSS, Inc., Chicago, IL, U.S.A.) to analyze research dates. The quantitative values were presented as mean ± SD, and the hypothesis test for significance between two groups utilized the Student’s *t*-test (two-tailed). *P* values <0.05 were considered statistically significant.

### Ethical approval

Ethical approval for the present study was obtained from the Ethics Committee of the First Affiliated Hospital of Wenzhou Medical University.

## Result

### LncRNA PROX1-AS1 was up-regulated in PTC

**Figure 1:**
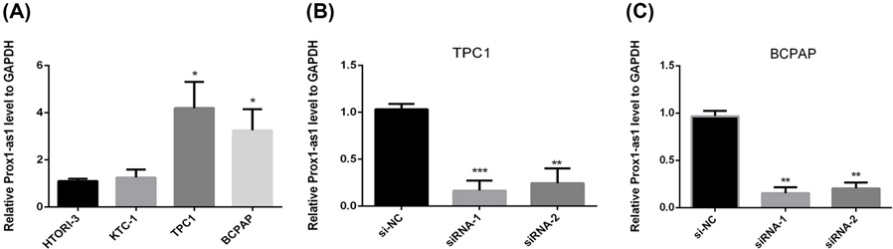
PROX1-AS1 has higher expression in PTC cell lines (**A**) The relative expression of PROX1-AS1 gene (compared with the GAPDH gene) was examined by qRT-PCR. Compared with the other thyroid cancer cell lines, PROX1-AS1 expressed a higher level in TPC1 and BCPAP cell lines. (**B** and **C**) The relative expression of PROX1-AS1 gene (compared with the GAPDH gene) in TPC1 and BCPAP. Compared with corresponding control group, the expression of PROX1-AS1 in siRNA groups was lower; *P<0.05, **P<0.01, ***P<0.001 in comparison with the controls using Student’s *t*-test.

To study the potential biological functions of LncRNA PROX1-AS1 in PTC cell lines, we examined the PROX1-AS1 expression levels in several PTC cell lines and normal thyroid cell line by qRT-PCR. As a result, PROX1-AS1 was found to be expressed higher in PTC cell lines compared with that in the normal thyroid cell line (Figure 1A). Among these, the expression level of PROX1-AS1 in TPC1 and BCPAP cell lines had statistical significance (*P*<0.05) (Figure 1B,C), so we utilized these two cell lines for the further experiments. This assay demonstrated that PROX1-AS1 was related to PTC and might function as an oncogene in PTC.

### LncRNA PROX1-AS1 promoted proliferation in PTC cell lines

**Figure 2:**
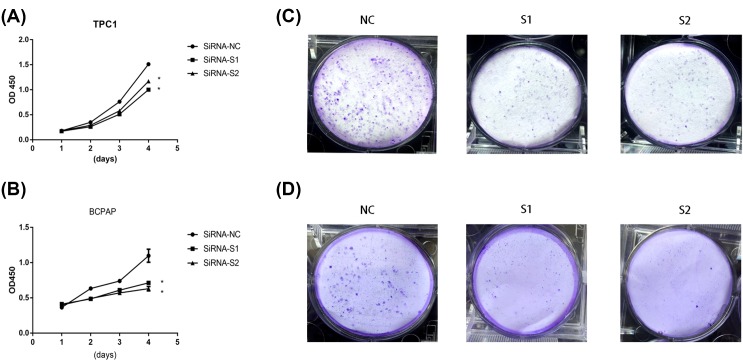
Down-regulation of PROX1-AS1 expression inhibits PTC cell lines proliferation (**A** and **B**) TPC1 and BCPAP cell lines transfected with si-RNA or si-NC were cultured in 96-well plates for 1–4 days, then measured cell proliferation via CCK-8 kits. Cell proliferation was significantly suppressed in TPC1 and BCPAP transfected by si-RNA. (**C** and **D**) TPC1 and BCPAP cell lines transfected with si-RNA or si-NC were cultured in six-well plates for 7–10 days. *P<0.05 in comparison with si-NC using Student’s *t*-test.

In the CCK-8 proliferation assay, down-regulation of PROX1-AS1 gene showed a significant inhibition of proliferation compared with the control group (Figure 2A,B). The similar consequence was also found in the colony formation assay where the colony numbers were decreased owing to the knocking down of the PROX1-AS1 (Figure 2C,D). In a word, these results indicated that highly PROX1-AS1 may promote proliferation in PTC cells.

### LncRNA PROX1-AS1 promoted invasion and migration in PTC cell lines *in vitro*

**Figure 3.**
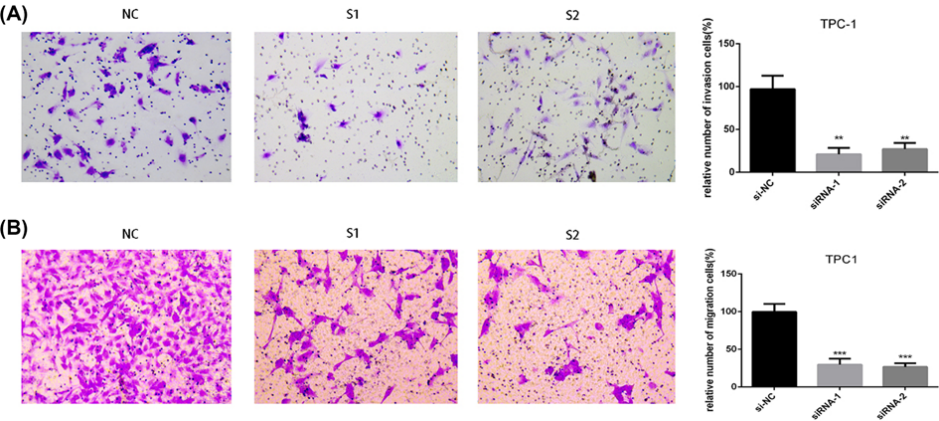
Down-regulation of PROX1-AS1 gene expression inhibits invasion and migration in TPC1 cell lines (**A**) Transwell invasion assay in down-regulation of PROX1-AS1 cells and their corresponding control cells. (**B**) Transwell migration assay in down-regulation of PROX1-AS1 cells and their corresponding control cells. Quantitative results of invasion and migration assays. The columns represent the mean of cell numbers from at least three independent experiments; **P<0.01, ***P<0.001 in comparison with the NC group using Student’s *t*-test.

**Figure 4:**
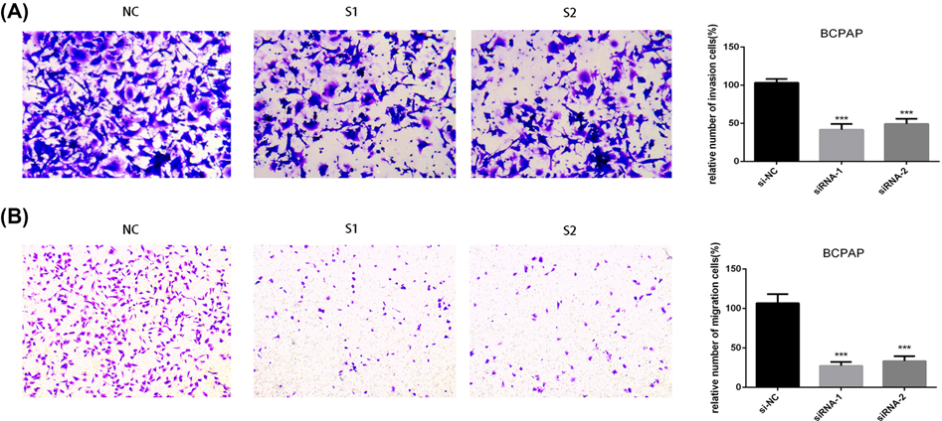
Down-regulation of PROX1-AS1 gene expression inhibits invasion and migration in BCPAP cell lines (**A**) Transwell invasion assay in down-regulation of PROX1-AS1 cells and their corresponding control cells. (**B**) Transwell migration assay in down-regulation of PROX1-AS1 cells and their corresponding control cells. Quantitative results of invasion and migration assays. The columns represent the mean of cell numbers from at least three independent experiments. ***P<0.001 in comparison with the NC group using Student’s *t*-test.

To validate the function of PROX1-AS1 on invasion and migration in PTC cells, we carried out the Transwell invasion assay where silencing PROX1-AS1 extremely decreased the invasion ability in both TPC1 and BCPAP cell lines (Figures 3A and 4A). Similar results were observed in the migration assay. Down-regulation of PROX1-AS1 expressed extremely lower migration potential in PTC cell lines contrasted with the controls (Figures 3B and 4B). Thus, these results replied that PROX1-AS1 could promote invasion and migration of PTC cells.

### LncRNA PROX1-AS1 might act on epithelial–mesenchymal transition (EMT) process

**Figure 5:**
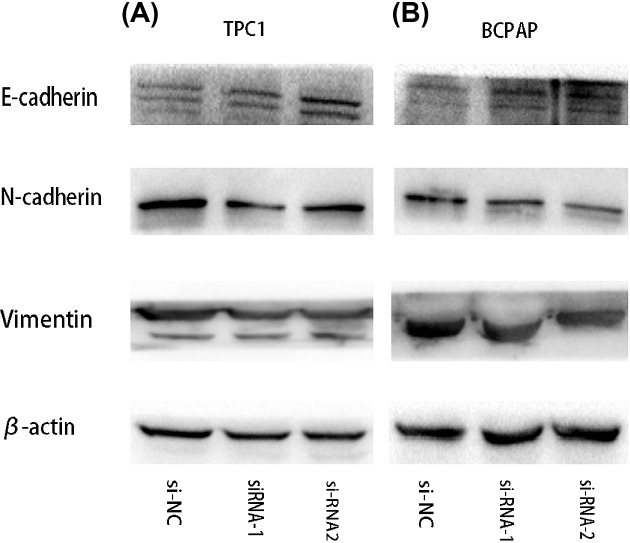
Down-regulation of PROX1-AS1 might regulate the invasion and migration of PTC cells via EMT (**A** and **B**) The effect of PROX1-AS1 expression on the levels of E-cadherin, N-cadherin, and Vimentin in PTC cell lines transfected with si-RNA or si-NC by Western blot.

Given that the epithelial–mesenchymal transition (EMT) takes effect in tumor progression, we evaluated if PROX1-AS1 had any influence on EMT process through detecting the expression levels of some EMT-related proteins by WB. As shown in Figure 5, down-regulation of PROX1-AS1 gene induced enhancement of E-cadherin expression, whereas N-cadherin and Vimentin were down-regulated. Thus, these findings indicate that down-regulation of PROX1-AS1 may inhibit the EMT progression in PTC cells.

## Discussion

Papillary thyroid carcinoma (PTC) arises more and more frequently in the views of the world [13,14]. Although we have a better knowledge of the molecular biology of PTC, the potential molecular mechanisms underlying PTC initiation and progress remain unclear.

Recently, accumulated evidence has shown that LncRNAs may be a group of new stars for the mechanism exploration in various types of cancers [15,16]. The dysregulation of LncRNA was closely related to the process of carcinogenesis, invasion, and metastasis of malignant tumor, for example, LncRNA HOTAIR caused genome-wide retargeting PRC2 to promote breast cancer invasiveness and metastasis [17]. LncRNA KIAA0125 also has been found that it could promote the progress of gallbladder carcinoma through modulating the expression of β-catenin and Vimentin [18]. To understand more about PTC, we found that LncRNA PROX1-AS1 was up-regulated in PTC through genome sequencing. However, few papers have reported the potential roles of PROX1-AS1 in the progression of cancer, especially in PTC.

In our study, we first examined the expression of PROX1-AS1 in papillary thyroid cancer cell lines compared with the controls. The results showed that PROX1-AS1 was significantly up-regulated both in TPC1 and BCPAP cell lines. Besides, silencing PROX1-AS1 significantly inhibited proliferation, colony formation, migration, and invasion of PTC cell lines *in vitro*. The above indicated that PROX1-AS1 acted as a promoter of PTC progress. Furthermore, we also showed for the first time that the function of PROX1-AS1 in PTC might be associated with the EMT. EMT is considered as a process where epithelial cells lose the ability of cell polarity and gain the skill of metastasis, and then convert to mesenchymal cells [19]. It is involved in the generation of tissues and organs during embryogenesis [20]. It has now been more than 10 years since EMT was first confirmed to be intimately associated with cancer progression [21,22]. In our study, we have found that knockdown of PROX1-AS1 could down-regulate the expression of N-cadherin and Vimentin, while E-cadherin was up-regulated. All of these proteins are EMT-related markers.

However, several drawbacks also existed in our study. We didn’t bind the LncRNA PROX1-AS1 with the clinic-pathological features of PTC due to lack of integral clinical data, and further experiments *in vivo* should be performed if possible. Thus, much work still needs to be fulfilled so as to deepen our knowledge about PTC. In summary, our study may provide a theoretical basis for the possibility of PROX1-AS1 to be a novel diagnostic and therapeutic target for papillary thyroid carcinoma.

## Abbreviations

CCK-8: Cell Counting Kit-8
EMT: epithelial–mesenchymal transition
LncRNA: long noncoding RNA
PTC: papillary thyroid carcinoma
